# Supplementary material for: Vitamin D Modified DSS-Induced Colitis in Mice via STING Signaling Pathway
Source: Biology (Basel). 2025 Jun 18;14(6):715. doi: 10.3390/biology14060715 (PMC12190092; doi:10.3390/biology14060715)
Supplement: Supplementary file 1 [file biology-14-00715-s001.zip › Supplementary Materials S1: 16s-report of gut microbiota/Groups/heatmap/nogroup/non_norm_100log10.pdf]

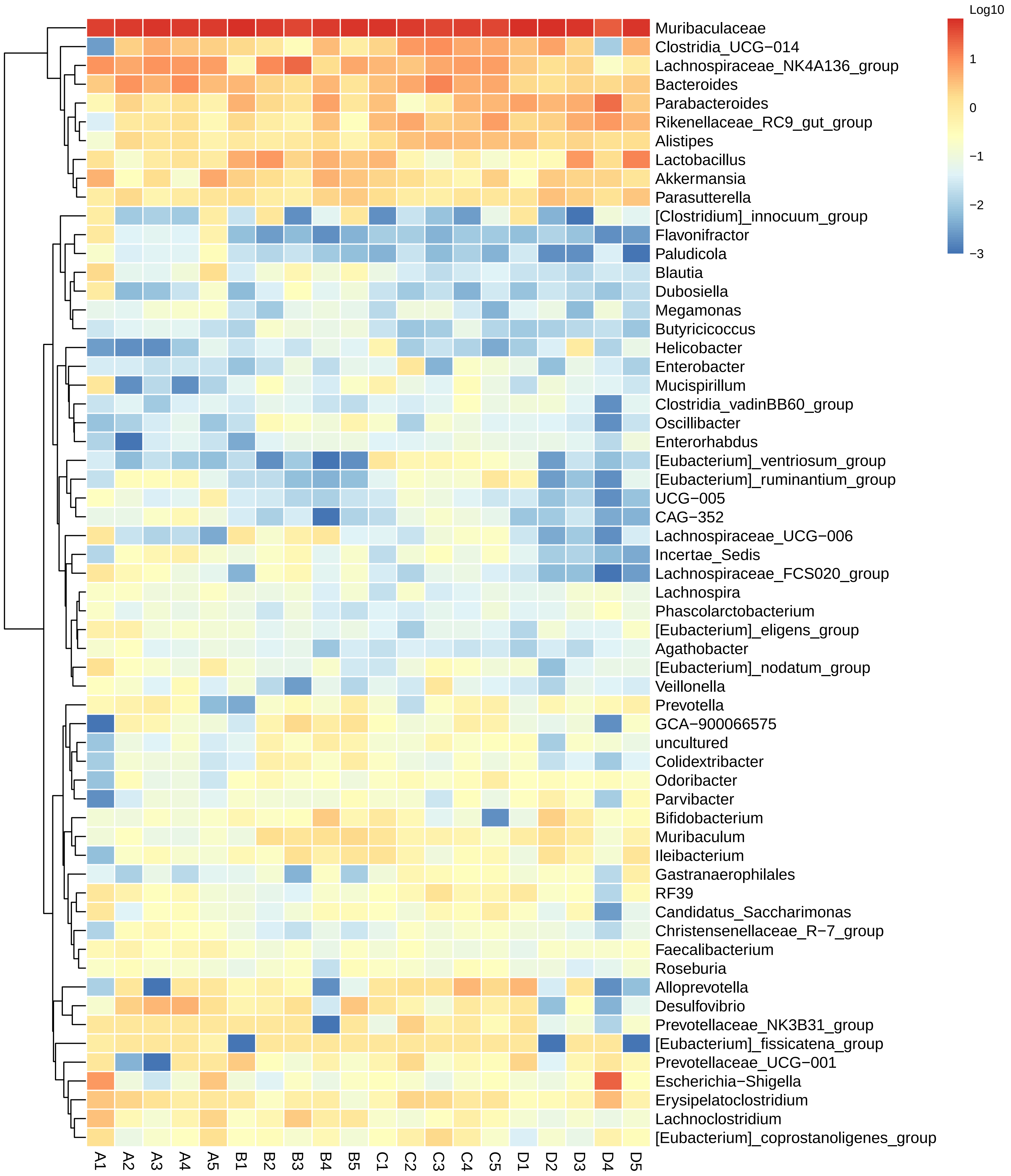

Log10
1
0
−1
−2
−3
Muribaculaceae
Clostridia_UCG−014
Lachnospiraceae_NK4A136_group
Bacteroides
Parabacteroides
Rikenellaceae_RC9_gut_group
Alistipes
Lactobacillus
Akkermansia
Parasutterella
[Clostridium]_innocuum_group
Flavonifractor
Paludicola
Blautia
Dubosiella
Megamonas
Butyricicoccus
Helicobacter
Enterobacter
Mucispirillum
Clostridia_vadinBB60_group
Oscillibacter
Enterorhabdus
[Eubacterium]_ventriosum_group
[Eubacterium]_ruminantium_group
UCG−005
CAG−352
Lachnospiraceae_UCG−006
Incertae_Sedis
Lachnospiraceae_FCS020_group
Lachnospira
Phascolarctobacterium
[Eubacterium]_eligens_group
Agathobacter
[Eubacterium]_nodatum_group
Veillonella
Prevotella
GCA−900066575
uncultured
Colidextribacter
Odoribacter
Parvibacter
Bifidobacterium
Muribaculum
Ileibacterium
Gastranaerophilales
RF39
Candidatus_Saccharimonas
Christensenellaceae_R−7_group
Faecalibacterium
Roseburia
Alloprevotella
Desulfovibrio
Prevotellaceae_NK3B31_group
[Eubacterium]_fissicatena_group
Prevotellaceae_UCG−001
Escherichia−Shigella
Erysipelatoclostridium
Lachnoclostridium
[Eubacterium]_coprostanoligenes_group
A1
A2
A3
A4
A5
B1
B2
B3
B4
B5
C1
C2
C3
C4
C5
D1
D2
D3
D4
D5
